# Polyphosphate in Neonates: Less Shedding from Platelets and Divergent Prothrombotic Capacity Due to Lower TFPI Levels

**DOI:** 10.3389/fphys.2017.00586

**Published:** 2017-08-24

**Authors:** Axel Schlagenhauf, Harald Haidl, Sina Pohl, Eva-Christine Weiss, Bettina Leschnik, Siegfried Gallistl, Wolfgang Muntean

**Affiliations:** ^1^Department of General Pediatrics and Adolescent Medicine, Medical University of Graz Graz, Austria; ^2^Department of Obstetrics and Gynecology, Medical University of Graz Graz, Austria

**Keywords:** newborn, polyphosphate, blood platelets, thrombin, blood coagulation tests

## Abstract

**Background:** The neonatal hemostatic system exhibits a fragile balance featuring lower levels of clotting factors as well as inhibitors. Neonatal platelets show *in-vitro* hypoaggregability, but neonates exhibit well-functioning primary and secondary hemostasis despite this impairment. Recently, polyphosphate shed by activated platelets has been shown to induce a prothrombotic shift on the plasmatic coagulation system of adults. The impact of platelet derived polyphosphate might differ in neonates due to aforementioned peculiarities.

**Aims:** We aimed to comparatively determine polyphosphate content and release from adult and neonatal platelets and to determine its impact on thrombin generation in plasma from adult and cord blood.

**Methods:** Polyphosphate was extracted from adult and neonatal platelet lysates and releasates using silica spin-columns and quantified with a DAPI based fluorescence assay. The impact of exogenous polyphosphate in various concentrations (208–0.026 μg/ml) on thrombin generation was evaluated in plasma from adult and cord blood as well as in adult plasma with reduced tissue factor pathway inhibitor (TFPI) levels using calibrated automated thrombography.

**Results:** Polyphosphate content was comparable in both groups, but the fraction of released polyphosphate upon stimulation with thrombin receptor activating peptide was lower in neonatal samples (adult: 84.1 ± 12.9%; cord: 58.8 ± 11.2%). Relative impact of polyphosphate on lag time of thrombin generation was higher in adult samples compared to samples from cord blood (adult: 41.0% [IQR: 35.2–71.8%] of vehicle; cord: 73.4% [IQR: 70.2–91.4%] of vehicle). However, in samples from cord blood, lower concentrations of polyphosphate were required to obtain maximal impact on thrombin generation (adult: 26 μg/ml; cord: 0.814 μg/ml). PolyP affected thrombin generation in adult plasma similarly to cord plasma, when the TFPI concentration was reduced to neonatal levels.

**Conclusion:** Differences in the impact of polyphosphate on adult and neonatal coagulation are largely caused by differences in TFPI levels. Lower polyphosphate release from neonatal platelets, but lower optimum concentration to drive neonatal plasmatic hemostasis emphasizes the well-matched, but fragile interplay between platelets and coagulation in newborns. A potential developmental mismatch should be considered when transfusing adult platelets into neonates.

## Introduction

Hemostasis of healthy full-term neonates exhibits peculiarities that cannot be observed in adults. Most characteristics of the neonatal hemostatic system are results from its development in the fetus over time (Andrew et al., 1992). The activated partial thromboplastin time (aPTT) is prolonged when compared to adult reference ranges. This is most likely attributed to the lower concentrations of coagulation factors after birth (Andrew et al., 1987). Despite this peculiarity, neonates show no increased bleeding during surgery and good wound healing, which is in contrast to results from *in-vitro* testing (Manco-Johnson, 2008). Our group showed that thrombin generation in neonates is facilitated by low levels of AT, protein C, and protein S as well as elevated levels of α2-macroglobulin (Cvirn et al., 2002). In addition, the concomitant action of low levels of tissue factor pathway inhibitor (TFPI) and antithrombin (AT) results in shorter clotting times as well as faster factor Xa and thrombin generation in cord as compared to adult plasma when small amounts of lipidated tissue factor (TF <10 pm) are applied to initiate clotting. The amount of free thrombin generated is 90% of adult levels (Cvirn et al., 2003).

The neonatal platelet phenotype differs from that of adults *in-vitro*, exhibiting hypoaggregability with many commonly used agonists such as ADP, epinephrine, thrombin, and thromboxane analogs (Mull and Hathaway, 1970; Israels et al., 1990; Rajasekhar et al., 1994). Platelet preactivation during accouchement and resultant refractoriness has been proposed, but in agreement with other research groups we found no increase in activation markers on neonatal platelets after delivery (Nicolini et al., 1994; Grosshaupt et al., 1997; Irken et al., 1998). Instead, multifactorial impairments in signal transduction of neonatal platelets have been shown to cause hyporeactivity. Neonatal platelets exhibit impaired calcium mobilization upon stimulation, lower numbers of α 2-adrenergic receptors, and lower GTPase activity in G_q_-coupled receptors accounting for reduced reactivity to various agonists (Corby and O'Barr, 1981; Gelman et al., 1996; Israels et al., 1999). Despite this known hyporeactivity, neonatal primary hemostasis *in vivo* seems to be functional (Andrew et al., 1990; Israels et al., 2001; Boudewijns et al., 2003).

Investigating the role of platelets in secondary hemostasis, we observed that neonatal platelets support thrombin generation as well as adult platelets (Bernhard et al., 2009). This is extraordinary because responsiveness to thrombin is necessary for the expression of negatively charged phospholipids on the platelet surface that is driving thrombin generation. We identified lower levels of protease-activated receptors 1 and 4 in neonatal platelets of cord blood as potential cause for their hypoaggregability to thrombin (Schlagenhauf et al., 2010). Furthermore, we showed that peak levels of prostaglandin E2 at birth provide a modest inhibitory effect on platelets in neonates, potentially protecting against preactivation during accouchement (Schlagenhauf et al., 2015). This effect is lost within the first hours of life. These findings argue for a neonatal platelet phenotype that is well-adapted to special requirements before, during and after birth, providing an optimal interplay with the neonatal plasmatic hemostatic system.

A new link between platelet activation and plasmatic hemostasis has been discovered. Inorganic polyphosphate (PolyP) is involved in thrombosis and inflammation. High molecular weight PolyP can induce contact activation via factor XII, thus, acting as trigger for the intrinsic pathway (Smith et al., 2006, 2010; Morrissey et al., 2012). Furthermore, PolyP accelerates thrombin generation by promoting factor XIa induced factor V activation, inhibiting TFPI, and enhancing the feedback loop of factor XI activation by thrombin (Choi et al., 2011, 2015).

It has been shown that PolyP with an average polymer size of 70–75 phosphate residues is stored in delta granules of platelets which are released upon platelet activation (Ruiz et al., 2004). Although this platelet-derived PolyP with medium chain length has been shown to induce minimal factor XII activation, the predominant prothrombotic stimulus is more likely to be mediated via TFPI inactivation and more rapid factor XI activation (Muller et al., 2009; Faxalv et al., 2013).

As mentioned above, thrombin generation in neonatal samples is lower than in adult samples at high tissue factor concentrations but comparable at low tissue factor concentrations which makes the assay more sensitive to inhibitory factors and positive feedback loops. Due to lower concentrations of clotting factors as well as inhibitors, the neonatal hemostatic balance is more fragile than that of adults. We therefore hypothesize that platelet-derived PolyP exhibits a higher prothrombotic potential in the neonate than in the adult. Higher disposition of the neonatal coagulation system to undergo a PolyP induced prothrombotic shift might compensate for reduced platelet shape change. However, the influence of PolyP on the neonatal plasmatic hemostasis needs to be evaluated in combination with its release from neonatal platelets, to take all developmental peculiarities into account.

Our aim was to determine the specific role of platelet-derived PolyP on the neonatal in contrast to the adult hemostatic system. For this purpose, we quantified PolyP content as well as release from neonatal and adult platelets. Furthermore, we determined the respective impact of exogenous PolyP on thrombin generation in plasma from cord blood and adult blood using calibrated automated thrombography.

## Materials and methods

### Materials

An intermediate-chain PolyP standard with polymer lengths of 40–160 and a modal size of 75 phosphate units was purchased from Kerafast (Boston, Massachusetts, USA). Prostaglandin I_2_ was purchased from Cayman (Ann Arbor, USA). Thrombin receptor activating peptide-6 was purchased from Multiplate (Multiplate Services GmbH, Munich, Germany). Silica spin columns and Qiagen protease were obtained from a QIAmp DNA blood mini kit (Qiagen, Hilden, Germany). PPP reagent, MP-reagent, and calibrator were purchased from Thrombinoscope (Stago, Vienna, Austria). All reagents for one-stage clotting assays were purchased from Stago (Vienna, Austria). TFPI ELISA, and TFPI depleted plasma was purchased from Sekisui Diagnostics (Pfungstadt, Germany). All other reagents were purchased from Sigma Aldrich (Vienna, Austria) if not stated otherwise.

#### Subjects and pre-analytics

This study was carried out in accordance with the recommendations of the Ethics Committee of the Medical University of Graz, Austria (EK-Nr. 28-428 ex 15/16) with written informed consent from all subjects. All adult subjects gave written informed consent in accordance with the Declaration of Helsinki. All mothers gave written informed consent for the procurement of cord blood. The protocol was approved by the Ethics Committee of the Medical University of Graz.

From 12 self-reported adult controls blood was drawn with a 21 gauge needle from the antecubital vein, without applying venostasis, into pre-citrated S-Monovette premarked tubes (3 ml) from Sarstedt, containing 0.30 ml 0.106 mol/l trisodium citrate solution. The adult cohort consisted of 6 females and 6 males with a median age of 36 years (range: 26–61 years). Cord blood was obtained from 17 healthy full-term neonates, following uncomplicated delivery after 39–42 weeks of gestation.

### Determination of clotting factor and inhibitor levels

Levels of clotting factors II, V, VII, VIII, IX, X, and XI were determined in all samples using routine one-stage clotting assays on a STA Compact Max 2 (Stago, Vienna, Austria). Antithrombin levels were determined with a routine chromogenic method. Total TFPI plasma levels were determined using ELISA.

#### Determination of platelet PolyP content and release

Platelet PolyP content and release was determined as reported previously (Schlagenhauf et al., 2016) in samples from adult blood (content: *N* = 12, release: *N* = 8) and from cord blood (content: *N* = 13, release *N* = 8). Briefly, for determination of platelet content, platelets were isolated from platelet-rich plasma by centrifugation and lysed by repetitive ultrasonic bursts in sample buffer (5 mM Tris, 100 mM NaCl, 5 mM EDTA, pH 7.4). For determination of PolyP, secretion platelets were resuspended in sample buffer before incubation with thrombin receptor activating peptide (TRAP, 32 μM) at 37°C under constant stirring for 15 min. Platelets were pelleted by centrifugation and the supernatant was used for further analysis.

Platelet lysates and supernatants were subjected to protease digestion according to manufacturer's instruction (56°C, 10 min), then mixed with 5.5 M GITC and ethanol (sample/GITC/ethanol, 1/3/8, v/v/v), respectively. Mixtures were loaded onto silica spin columns, washed twice with wash buffer (sample buffer/ethanol, 1/1, v/v), and eluted with 5 × 90 μl sample buffer. Elutions were diluted to 1 ml with sample buffer, and PolyP-content was detected by fluorescence measurements using 50 μM 4',6-diamidino-2-phenylindole (excitation 415 nm, emission 550 nm). PolyP quantification was done with external standardization using various dilutions of a PolyP standard.

### Calibrated automated thrombography

Thrombin generation was performed according to Hemker et al. as reported previously (adult: *N* = 12, cord: *N* = 17) (Hemker et al., 2003; Schweintzger et al., 2011). The final reaction mixture contained 0.5 pM tissue factor and 4 μM phospholipids. Exogenous PolyP was added in 1:1 serial dilutions with final concentrations ranging from 0.026 to 208 μg/ml. Lag time, time to peak, peak height, and the endogenous thrombin potential (ETP) were recorded. The velocity index was calculated with the formula [(time to peak—lag time)/peak height].

The same procedure was done with a mixture of standard plasma and TFPI depleted plasma (final concentration: 15.37 ng/ml TFPI) in comparison with adult standard plasma (48.33 ng/ml TFPI). These experiments were done in triplicates.

To evaluate the polymer-specific effects of PolyP on thrombin generation, various dilutions of PolyP were digested with phosphatase from calf mucosa (0.05 U/μg PolyP, 37°C, 8 h under shaking). Quantitative digestion was controlled by aforementioned fluorescence measurements. Digested samples were tested in CAT in addition to undigested dilutions.

#### Statistics

All calculations were done using Graphpad Prism 6.0 (Graphpad software, San Diego, CA). Values are given as mean ± standard deviation or median [IQR]. Student's *t*-test for normally distributed data and Mann-Whitney *U*-test for not normally distributed data were applied to analyze differences in parameters. Corrections for multiple testing at different PolyP concentrations were performed with the Holm-Šídák method (α = 0.05), and multiplicity adjusted *P*-values were calculated for each comparison.

## Results

### Clotting factor and TFPI levels in samples from cord blood and adult blood

Samples from cord blood exhibited significantly lower levels of factor II, VII, IX, XI, anthrombin, and TFPI than samples from adult blood (Table 1).

**Table 1:** Clotting factor and inhibitor levels in cord blood and adult blood samples (mean ± sd).

	**Adult**	**Cord**
Factor II [%]	94.3 ± 11.4	54.3 ± 6.5^***^
Factor V [%]	87.5 ± 13.7	87.0 ± 5.5
Factor VII [%]	91.3 ± 15.8	77.3 ± 8.4^*^
Factor VIII [%]	96.2 ± 13.3	94.7 ± 8.2
Factor IX [%]	101.4 ± 5.9	46.9 ± 9.4^***^
Factor X [%]	87.4 ± 10.1	91.6 ± 9.4
Factor XI [%]	119.5 ± 38.0	40.7 ± 5.3^***^
Antithrombin [%]	107.8 ± 12.5	53.0 ± 7.7^***^
TFPI [ng/ml]	48.3 ± 20.4	15.4 ± 6.6^***^

Differences from respective adult parameters

*** *P < 0.001*,

* *P < 0.05*.

#### PolyP content and release from neonatal and adult platelets

**Figure 1:**
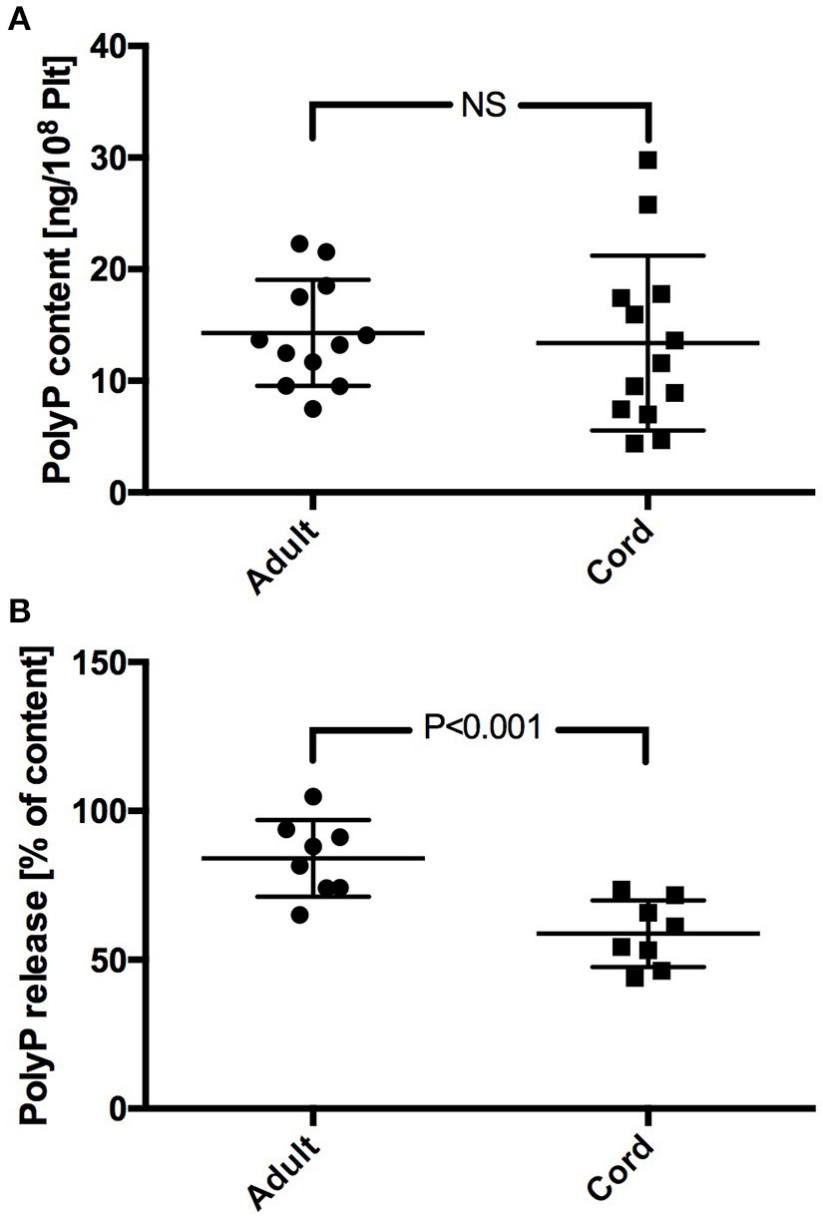
PolyP content **(A)**, and percentage of PolyP released upon stimulation with TRAP **(B)** in neonatal and adult platelet samples.

Platelet PolyP content scattered largely in both study groups. No significant difference was found in PolyP content between adult and neonatal platelets (adult: 14.3 ± 4.8 ng/10^8^ Plt; cord: 13.4 ± 7.8 ng/10^8^ Plt) (Figure 1A). PolyP release from platelets upon activation with TRAP was lower in samples from cord blood. Calculation of the respective released fractions revealed a significantly lower percentage in samples with neonatal platelets (adult: 84.1 ± 12.9%; cord: 58.8 ± 11.2%; *P* < 0.001) (Figure 1B).

### Influence of PolyP on thrombin generation

#### Differences between cord blood and adult blood

**Figure 2:**
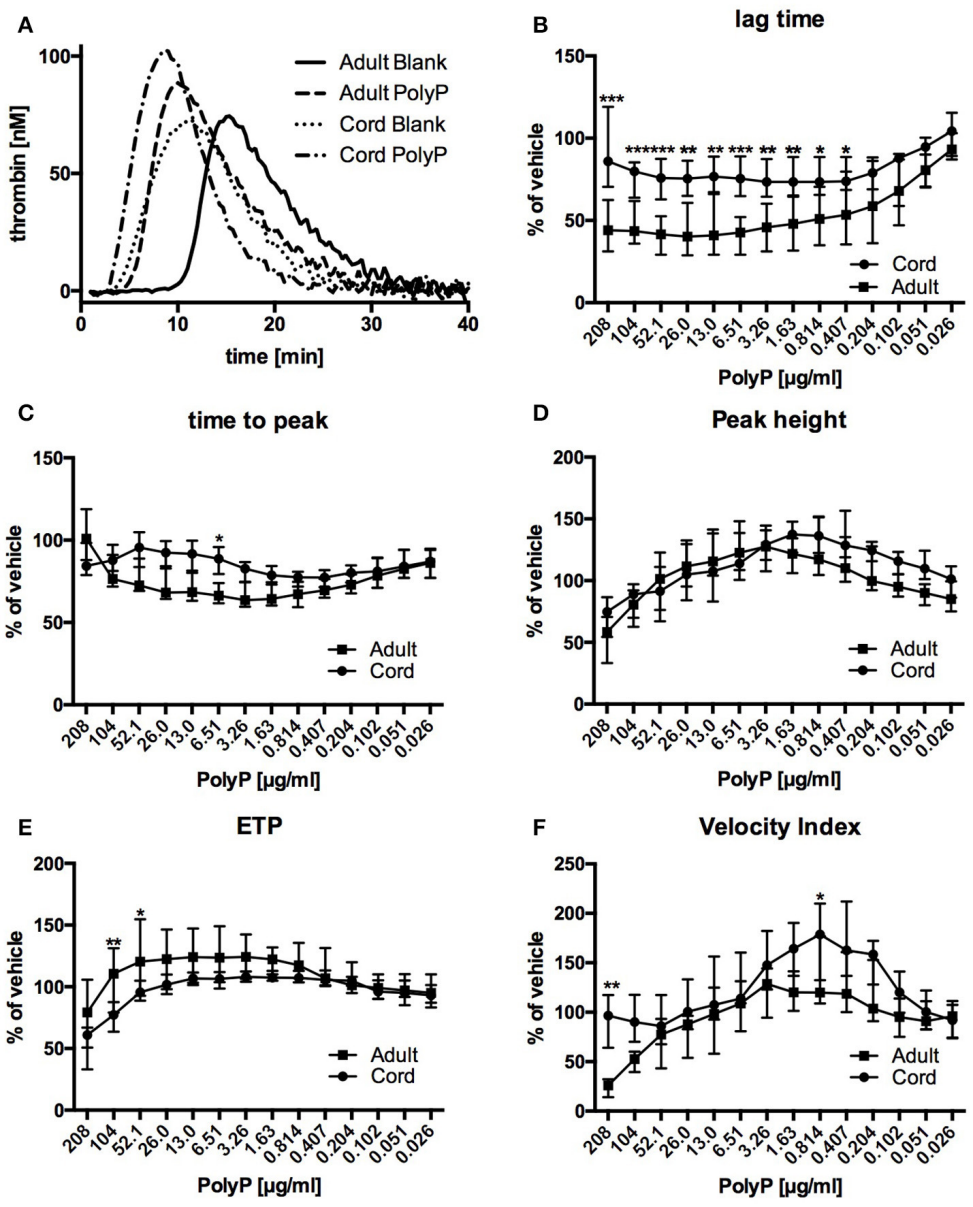
Representative thrombin generation traces **(A)** and relative influence of PolyP on respective CAT-parameters in plasma from cord and adult blood **(B–F)**. Data are depicted in % of vehicle and median (IQR). ^***^P < 0.001, ^**^P < 0.01, ^*^P < 0.05.

Thrombin generation traces of both, samples from adult blood as well as samples from cord blood, were substantially changed by addition of PolyP (Figure 2A).

Lag time without PolyP was longer in plasma from adult blood than in plasma from cord blood (Table 2). Influence of PolyP on thrombin generation differed in the two study groups (Figure 2A). PolyP shortened the lag time in plasma from adults in a concentration range of 13.0–208 μg/ml. and in plasma from cord blood in a concentration range of 0.407–104 μg/ml (Figure 2B). At respective optimum PolyP concentrations (adult: 26 μg/ml; cord: 0.814 μg/ml), lag time in adult samples was shortened to 41.0% [IQR: 35.2–71.8%] of vehicle; in cord samples to 73.4% [IQR: 70.2–91.4%] of vehicle.

**Table 2:** Absolute CAT-parameters without PolyP and at the respective optimum PolyP-concentration.

	**Adult**		**Cord**	
	**Vehicle**	**PolyP**	**Vehicle**	**PolyP**
Lag time [min]	10.22 [8.055–12.59]	4.000 [3.835–5.055]^**^	4.820 [4.185–6.165]^§§^	3.670 [3.465–3.895]^**^^§^
TTPeak [min]	15.17 [12.56–17.31]	9.890 [8.805–10.39]^**^	10.78 [10.06–12.86]^§§^	8.670 [8.000–9.385]^**^^§^
Peak height [nM]	75.63 [55.27–85.41]	91.85 [66.41–105.6]^*^	75.54 [68.37–88.55]	106.3 [91.73–122.3]^**^
ETP [nM^*^min]	707.3 [597.3–862.0]	899.3 [780.4–1158]^*^	774.8 [647.2–889.0]	863.8 [778.5–923.8]^**^
Vel. Index [nM/min]	14.81 [12.20–21.07]	18.56 [13.22–29.57]	11.07 [9.915–15.24]	20.42 [17.64–24.91]^**^

*Data are depicted in median [IQR]*.

Differences from respective vehicle;

* P < 0.05;

** P < 0.001;

Differences from respective adult parameters;

§ P < 0.05;

§§ *P < 0.001*.

Time to peak without PolyP was longer in plasma from adult blood than in plasma from cord blood. The optimum concentration for maximum reduction was substantially lower in cord samples (adult: 3.26 μg/ml; cord: 0.814 μg/ml), but the PolyP mediated relative reduction of time to peak was higher in adult samples (adult: 66.1% [IQR: 62.4–73.8%] of vehicle; cord: 76.7% [IQR: 71.6–77.2%] of vehicle) (Figure 2C).

Peak thrombin generation without PolyP was comparable in adult and cord samples. The optimum PolyP concentration to influence peak thrombin generation was lower in cord samples (adult: 3.26 μg/ml; cord: 1.63 μg/ml). Relative influence of PolyP on peak height was not significantly different in both study groups (adult: 127.6% [143.7–113.8] of vehicle; cord: 137.5% [119.7–146.3%] of vehicle) (Figure 2D).

The overall amount of thrombin (endogenous thrombin potential, ETP) that could be triggered without PolyP was comparable in both study groups. The optimum concentration of PolyP to influence the ETP was lower in samples from cord blood (adult: 3.26 μg/ml; cord: 0.814 μg/ml). PolyP increased ETP only to a small degree, and there was no significant difference between the relative influence of PolyP (adult: 124.3% [IQR: 112.5–138.1%] of vehicle; cord: 108.1% [IQR:106.9–112.7%] of vehicle) (Figure 2E).

Without PolyP there was no significant difference in the velocity index of both study groups. The optimum concentration of PolyP to influence the velocity index was lower in samples from cord blood (adult: 3.26 μg/ml; cord: 0.814 μg/ml), but the relative influence of PolyP was significantly higher in samples from cord plasma (adult: 128.7% [IQR: 85.4–141.1%] of vehicle; cord: 178.6% [IQR: 131.5–213.7%] of vehicle) (Figure 2F).

### Influence of PolyP at different TFPI concentrations

**Figure 3:**
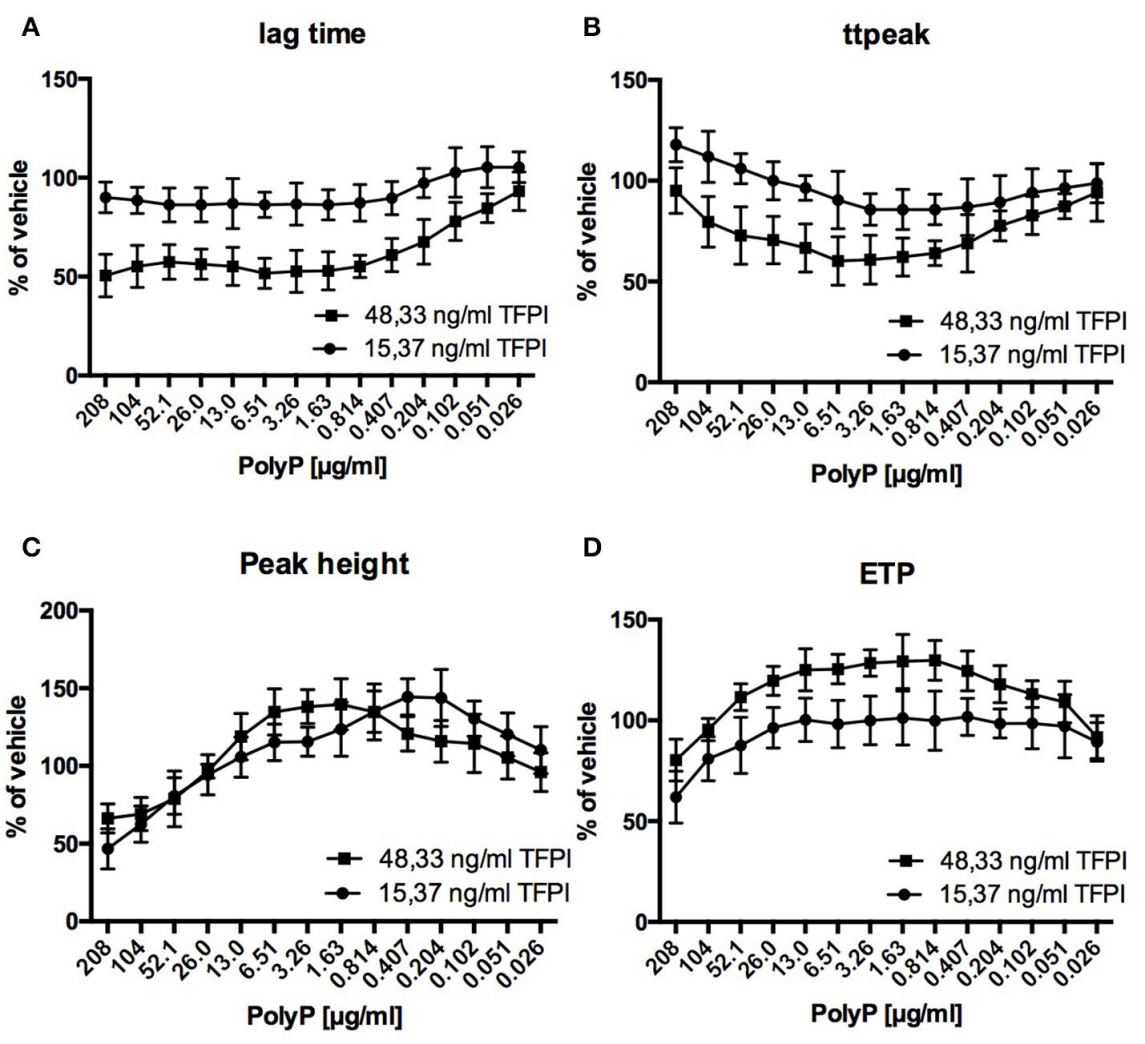
Relative influence of PolyP on respective CAT-parameters in standard adult plasma (48.33 ng/ml TFPI) and TFPI reduced plasma (15.37 ng/ml TFPI) **(A–D)**. Data are depicted in % of vehicle and mean ± sd.

Regarding the impact of PolyP on thrombin generation, adult plasma with reduced TFPI levels (15.37 ng/ml) differed significantly from adult standard plasma with normal TFPI levels (48.33 ng/ml) and was similarly affected as plasma from cord blood (Figure 3).

Relative reduction of lag time and time to peak was lower in TFPI reduced plasma compared to normal plasma (lag time: 86.3 ± 8.6 vs. 51.7 ± 7.6% of vehicle; time to peak: 85.7 ± 7.5 vs. 60.1 ± 12.0% of vehicle). Optimum PolyP concentrations for maximum impact on respective thrombin generation parameters were consistently lower in TFPI reduced plasma compared to normal plasma (lag time: 0.814 vs. 6.61 μg/ml; time to peak: 0.814 vs. 6.51 μg/ml; peak height: 0.407 vs. 1.63 μg/ml; ETP: 0.407 vs. 0.814 μg/ml).

#### Influence of digested PolyP

In contrast to undigested PolyP, digested PolyP did not influence parameters of thrombin generation at concentrations below 104 μg/ml (data not shown). At the highest concentrations (208 and 104 μg/ml) ETP and peak thrombin generation were decreased compared to vehicle, which was in line with results from undigested PolyP. These differences were observed with plasma from adult blood as well as from cord blood.

## Discussion

It has long been known that PolyP is a molecule with various biological functions that are established in several cells and tissues (Kumble and Kornberg, 1995; Kornberg, 1999), but only recent advances revealed the prothrombotic impact of platelet derived PolyP on plasmatic hemostasis (Muller et al., 2009; Morrissey et al., 2012).

In this study, PolyP content in platelets and its release upon stimulation was quantified for the first time in adult and neonatal samples. The inter-individual PolyP content in platelets varied significantly as reported previously (Ghosh et al., 2013). Although neonatal samples showed a slightly lower mean PolyP content, the two groups did not differ significantly. The fraction of PolyP released upon stimulation with TRAP in the adult group was similar to previously reported values (Ruiz et al., 2004) but lower in the neonatal group. This might be attributable to the well-known impaired release of dense granules from neonatal platelets (Whaun, 1980; Mankin et al., 2000). Although peak platelet aggregation response was obtained with this TRAP concentration (data not shown) we cannot exclude incomplete degranulation due to aforementioned impaired calcium mobilization and other signaling deficiencies (Corby and O'Barr, 1981; Gelman et al., 1996; Israels et al., 1999). On the other hand, a recent study demonstrated lower numbers of dense granules in neonatal platelets which might account for the lower PolyP release (Urban et al., 2017). It is conceivable that PolyP is distributed differently in neonatal platelets considering comparable PolyP content despite decreased dense granule numbers. Independent of the underlying cause, our data suggest that the amount of secreted PolyP potentially influencing plasmatic coagulation after platelet activation is lower in neonates than in adults.

Based on our quantifications in both groups at normal platelet counts, the amount of PolyP that would be released into the blood stream is between the lowest two concentrations tested in thrombin generation experiments. At this concentration the influence of PolyP is quite weak, and no differences between adult and cord samples were observable. However, local concentrations of PolyP accumulating within a platelet-rich clot would be substantially higher, thus providing a significant boost to thrombin generation.

The potential of PolyP to drive thrombin generation was highest when triggered with very low tissue factor concentrations (0.5 pM). Peak thrombin generation and ETP in both groups were comparable under these conditions, which is in agreement with previous observations (Cvirn et al., 2003). Measurement of thrombin generation over a range of PolyP concentrations revealed a U-shaped dose-response curve for most parameters in both study groups. We postulate that above the optimum concentration, the increase of ionic strength caused by the highly anionic PolyP leads to non-specific enzyme inhibition resulting in impaired thrombin generation. This theory is supported by the fact that addition of digested PolyP resulted in inhibition at higher concentrations but no PolyP-specific promotion of thrombin generation at lower concentrations.

Overall, plasma from cord blood exhibited a higher disposition to be influenced by low PolyP concentrations than plasma from adults. Differences in the optimum PolyP-concentration were observed in all thrombin generation parameters. The highest difference was observable in the lag time: Thrombin generation in adult samples revealed an optimum concentration of 26 μg/ml; cord samples had an optimum PolyP concentration of 0.814 μg/ml. However, the relative reduction of lag time induced by PolyP at the respective optimum concentration was substantially higher in adult samples compared to cord samples. Time to peak showed a similar picture. As a result, lag time and time to peak without PolyP are quite different in samples from adult and cord blood but are converging under PolyP influence.

We hypothesized that the observed differences in lag time and time to peak are largely caused by different TFPI levels. TFPI is the predominant factor influencing dynamic parameters (lag time, time to peak) and is known to be inhibited by PolyP (Smith et al., 2006). Inhibition of lower TFPI levels in neonates would need less PolyP, explaining the lower optimum PolyP concentrations in cord samples. On the other hand, lower TFPI levels in neonates also result in less “inhibition potential” for polyphosphate, explaining lower relative lag time changes in cord samples.

We tested this hypothesis by reducing TFPI levels in an adult reference sample to neonatal levels. As a result, the influence of PolyP was similar to its influence in cord samples. Lag time and time to peak were shortened at lower optimum PolyP concentrations, but the relative reduction was less compared to the adult sample with normal TFPI levels. PolyP may also accelerate FXI activation by thrombin (Choi et al., 2011), but reduction of FII and FXI did not change the influence of PolyP on thrombin generation (data not shown). We concluded that differing TFPI levels are the predominant cause for the diverging influence of PolyP on thrombin generation in adult and cord samples. The lower TFPI levels in cord samples match well with the lower PolyP release from neonatal platelets. Possibly, differing release of platelet derived PolyP and differing influence on adult and neonatal thrombin generation equalize hemostasis in both groups, resulting in comparable coagulation *in-vivo*.

We used direct fluorescence detection for determination of platelet PolyP content and release instead of indirect detection of orthophosphate release after incubation with recombinant exopopolyphosphatase. PolyP content in our samples was lower than reported previously (Ruiz et al., 2004; Hernandez-Ruiz et al., 2009; Ghosh et al., 2013). Since our method featuring PolyP enrichment on silica spin-columns showed quantitative recovery from platelet lysates (Schlagenhauf et al., 2016), we hypothesize that indirect detection of orthophosphate might have resulted in overestimation due to a high abundance of other potential orthophosphate-sources in the matrix.

A limitation of this study is the use of cord blood which is not representable for neonatal blood in all aspects because of differences in the placental endothelial layer. However, the platelet phenotype in cord blood has been shown to be comparable to that of platelets from neonatal blood within the first 24 h of life (Sitaru et al., 2005). Obtaining ample amounts of peripheral blood from healthy newborns was not ethically justifiable.

## Conclusion

Taken together, our data show a lower PolyP-release by neonatal platelets, but a higher disposition of plasma from cord blood to undergo a prothrombotic shift at low PolyP concentrations. Hence, we suggest a similar thrombotic impact of endogenous platelet derived PolyP in neonates and adults. The diverging PolyP-sensitivity in the two study cohorts is largely caused by differing TFPI levels and shows another aspect of the well-matched interplay between neonatal platelets and plasmatic hemostasis. Our findings shed a new light on neonatal hemostasis and emphasize the previously reported developmental mismatch of adult platelet transfusions with neonatal plasmatic coagulation (Ferrer-Marin et al., 2011). The increased PolyP secretion from adult platelets is not physiological in newborns and results in a higher thrombotic impact. PolyP-inhibitors have been suggested to reduce thrombosis while sparing hemostasis (Travers et al., 2014; Zhu et al., 2015), but the observed peculiarities should be taken into account when considering PolyP as potential therapeutic target in newborns.

This study investigated the role of platelet derived PolyP in healthy full-term newborns. Future studies will have to investigate the thrombotic potential of PolyP in pre-term newborns exhibiting an even higher hemostatic fragility than full-term newborns. Furthermore, the involvement of exogenous microbial PolyP in various neonatal pathologies should be investigated as it is conceivable that the release of microbial PolyP upon bacterial infection has a more deleterious effect due to its stronger prothrombotic potency in the newborn.

## What is known on this topic

- Dense granules of platelets contain polyphosphate, which is released upon platelet activation.
- Platelet derived polyphosphate induces a prothrombotic shift in plasmatic hemostasis.

## What this paper adds

- Polyphosphate content in adult and neonatal platelets is comparable, but neonatal platelets shed less polyphosphate upon activation.
- Plasma from cord blood exhibits a higher disposition to undergo a prothrombotic shift at low PolyP concentrations.
- These differences in PolyP sensitivity are predominantly caused by lower TFPI levels in samples from cord blood.

## Author contributions

All authors have made significant contributions in data collection, analysis, and interpretation of the findings. All co-authors have been involved in writing the final version of the manuscript.

### Conflict of interest statement

The authors declare that the research was conducted in the absence of any commercial or financial relationships that could be construed as a potential conflict of interest.
